# Reminiscences of Buda Pesth and the International Congress of Hygiene

**Published:** 1894-09-29

**Authors:** 


### REMINISCENCES OF BUDA-PESTH AND THE INTERNATIONAL CONGRESS OF HYGIENE.

(By an Occasional Correspondent.)

A juster idea is often gained in restrospect, rather than *au grande milieu*, of so colossal a meeting as that which recently occurred at Buda-Pesth to discuss from the international standpoint questions of hygiene and demography; and some impressions of a "congressist" (cleaving to him after his return home) may, perhaps, be worth recording. These impressions naturally range themselves under the heads of (1) the locale, (2) the *personnel*, (3) the work of the congress.

First, with regard to the locale, it would have been difficult to fix on a more pleasing meeting-place than Buda-Pesth. On the one side of the swift flowing Danube stands the old-world fortress city of Buda, the rock citadel of which has more than once served to arrest the flood of Eastern barbarism which threatened to swamp the civilisation of Europe. On the other side is the Parisian Pesth, with its quays, its cafés, and its boulevards. Here, in the extensive buildings of the Polytechnic School, was the congress installed. Much care and forethought had been exercised for the comfort of the members; for each was provided in a long gallery, under the initial of his name, a "post bag," in which each morning he found his daily journal, and ought also to have found his letters and invitations. Thus forewarned of the day's proceedings, the next task—and by no means a simple one—was to make tracks for the appropriate meeting-room. As the business was carried on in no less than twenty-six sections, sitting simultaneously, the chance of a good audience was but small, unless the subject under discussion was of more than ordinary interest. The great debates on cholera and diphtheria were crowded, and those in the children's section well attended, but in some other sections it was difficult to get a quorum. The general or plenary meetings held in various rooms of assembly somewhat distant from the Polytechnic were, with the exception of the inaugural one, but indifferently attended.

Passing now to the *personnel* of the congress, there was evidence of perhaps excessive centralisation in the hands of the general secretary. Most praiseworthy was the energy displayed by Dr. Colomar Müller, but it was beyond the power of any one man to satisfactorily meet the simultaneous demands of 2,500 congressists. More departmental organisation under responsible heads would have greatly facilitated smooth working; and national bureaux, with secretaries *en rapport* with the general secretary, would have been helpful to those who from ignorance of language or modes of procedure found themselves in difficulties. Of the willingness to oblige and intense energy of Dr. Müller and his assistants there was ample evidence; but the efforts of two or three officials amongst as many thousand visitors were necessarily inadequate.

With regard to the work of the congress, the risk of the flame of scientific discussion being dulled (if not smothered) by the mass of contributory fuel seemed great. Over 800 papers (in the four official languages) were announced; and it was only by the expedient of distributing them amongst twenty-six sections that all could be dealt with. As compared with the London congress of 1891, the great drawback was the absence of official abstracts. One had at Buda-Pesth solely the title of the paper itself to guide one, and this only in the language of the writer, which, if it happened to be Magyar, was intelligible only to a very limited section of the congress. Another unsatisfactory result of official abstracts not having been required was that in many instances papers announced to be read had actually never been written, the accredited authors having contented themselves with the advertisement which the publication of their names in the agenda afforded. The press also were much inconvenienced by not knowing beforehand the gist of each dissertation, and were driven to appeal directly to authors for this information. The multiplicity of sections added to the difficulty of reporting, but it is wonderful how well, notwithstanding, the proceedings have been reported in the leading medical journals. The linguistic drolleries of the sections were sometimes diverting, as, for instance, when a Hungarian president invited discussion on English papers in the complimentary formula, "Has man any remarks to *do* over the excellent lecture of Mr. So and So?" But what really was wanted to facilitate discussion was the publication in the four official languages of an abstract of each paper.

The subjects discussed have already been so well reported that it would be superfluous to enlarge on them. A word, however, may be said in commendation of the *entente cordiale* which prevailed between members of the medical and of the architectural and engineering professions present at the congress, which promises well for the practical house sanitation of the future. Educationalists also were well represented, and the growing interest in the hygienic side of education was satisfactorily shown by the large attendances in the section devoted to children. The report on the physical and mental condition of 50,000 school children, presented by the London committee, and the papers and demonstrations connected therewith, were specially well received.

The hospitalities lavished on the congress were profuse, and the danger of this Congress of Hygiene assuming too much the character of a gigantic pic-nic is one which the organisers of future meetings will do well to bear in mind. Thursday was given up to excursions, and those who had the good fortune to be the guests of Count Nicholas Esterházy at his estate of Tata (where he is the proprietor of some 180,000 acres) will not soon forget the splendid welcome they received at the hands of that popular Hungarian nobleman. Breakfast, luncheon, dinner, inspection of the stables and of the antiquities of the castle, a drive to the vineyards, a theatrical performance, and finally a dance, were some of the incidents of this visit, and it is whispered that some belated English guests who lost their way to the station were mistaken for burglars by the occupants of a house where they sought to make inquiries as to their route and were fired upon, fortunately, however, without effect, thus narrowly escaping the necessity of a more extended sojourn in the territories of the hospitable count. The Archduke, the Prime Minister, and the municipality also gave receptions, which were very numerously attended, and added much to the *éclat* of the meeting.

